# Pathogenicity and pathogenesis of a recent highly pathogenic avian influenza subtype H5N8 in mule ducklings in Egypt

**DOI:** 10.14202/vetworld.2023.59-67

**Published:** 2023-01-10

**Authors:** Mahmoud M. Abotaleb, Ahlam Mourad, Esraa Fouad, Walied Abdo, Samir A. Nassif

**Affiliations:** 1 Central Laboratory for Evaluation of Veterinary Biologics, Agriculture Research Center (ARC), Cairo, Egypt; 2 Department of Pathology, Faculty of Veterinary Medicine, Kafr-Elsheikh University, Egypt

**Keywords:** pathogenicity, pathogenesis, vaccination

## Abstract

**Background and Aim:**

In late 2017, an H5N8 highly pathogenic avian influenza (HPAI) virus, clade 2.3.4.4, was isolated from domestic ducks in Egypt, which was associated with high morbidity and low mortality. The pathogenicity increased due to the continuous circulation of virus in ducks. Thus, this study aimed to monitor the pathogenesis and pathogenicity of new H5N8 Avian influenza (AI) virus in mule ducklings.

**Materials and Methods:**

The lethal dose 50 (LD_50_) for this new local HPAI H5N8 isolate was calculated. Twenty ducklings were inoculated with 0.1 mL of dilution containing 10 LD_50_ HPAI per duck. The clinical signs and mortalities were recorded until 30 days post-infection (DPI) to confirm viral pathogenesis. Reverse transcription polymerase chain reaction was used to detect viral shedding from collected cloacal swabs after 3^rd^, 5^th^, 7^th^, 10^th^, 14^th^, 21^st^, and 30^th^ DPI. The main histopathological lesions associated with the presence of HPAI virus were also recorded on the 3^rd^ and 14^th^ DPI.

**Results:**

The result showed that the LD_50_ of the new HPAI H5N8 was 10^4^ log_10_. Clinical signs were observed after 2^nd^ DPI, but it was clinically severe on 3^rd^, 4^th^, and 5^th^ DPI in the form of respiratory and gastric disorders, forming 90% of all diseased ducklings, whereas 30% of the infected ducks only showed nervous signs. The mortality rate peaked on 4^th^ and 5^th^ DPI with a cumulative mortality rate of 60% for the inoculated ducks, whereas no mortality was recorded after 6^th^ DPI. Dead ducks showed typical postmortem lesions of AI disease. Necrosis and ecchymotic or petechial hemorrhages on the heart, pancreas, liver, and spleen were observed, whereas the lung showed pneumonia. With regard to viral shedding, infected ducklings shed the virus from its gut until 7^th^ DPI, but the number of duck shedders gradually decreased until 14^th^ DPI after viral shedding. The histopathological findings indicated that the spleen and thymus showed necrosis and hemorrhages, whereas the brain showed multifocal malacic foci and spread meningitis. Moreover, the lung had intrabronchial hyaline degeneration and fibrinous pneumonia on 3^rd^ DPI. Furthermore, the liver showed multifocal necrotic foci and subcapsular hemorrhage, whereas the kidney showed remarkable tubular degeneration, mostly within the collecting tubules. Furthermore, the heart showed marked myocardiolysis of the cardiac muscle fibers. On 14^th^ DPI, all histopathological lesions of the examined organs were restored to normal.

**Conclusion:**

The currently circulating HPAI H5N8 virus strain has high virulence, particularly for imported mule ducks that originated from non-vaccinated breeder ducks. Therefore, vaccination and quarantine measures must be applied on imported 1-day-old mule ducklings. Moreover, the pathogenesis must be reviewed and monitored for updating circulating AI strains caused by the continuous and rapid evolution of AI viruses.

## Introduction

Avian influenza (AI) virus can infect a wide range of domestic poultry under natural and experimental conditions, and its susceptibility varies on the basis of bird species [1]. Since 2006, the highly pathogenic (HP) AI subtype H5N1 virus clade 2.2.1 has been reported in domestic and wild birds in Egypt. Thus, authorities have directed the use of long vaccination. Despite these efforts, the H5N1 virus became enzootic, and the virus evolved into three antigenically distinct subclades (2.2.1.1, 2.2.1.1a, and 2.2.1.2). Recently, H5N8 HPAI virus of clade 2.3.4.4 was introduced to Egypt through migratory birds in 2016 [2]. In 2017, the same lineage of the virus was isolated from domestic ducks [3]. Waterfowl serves as the natural reservoir for AI viruses. Wild and domestic waterfowl have played an important role in the maintenance and spread of H5 HPAI viruses. Infected migratory waterfowls contribute to the spread of H5N1 and H5N8 HPAI viruses from Asia to other parts of the world.

The HP H5N1 virus was initially isolated from a farmed goose in Guangdong, China, in 1996, and then it spread worldwide. First, no mortality was reported among ducks infected with HPAI subtype H5N1, but they showed low morbidity and few viral shedding for 1–3 days after infection. Afterward, the virulence of the virus had remarkably increased and caused high morbidity and mortality in duck farms [2]. Clinical signs were observed in ducks infected with HPAI H5N8 virus, including respiratory manifestation, depression, and nervous signs such as torticollis and other unusual positions of the head. Congestion of the lung, spleen, and liver as well as congestion and edema of the subcutaneous tissue of the head and meninges were also observed. Moreover, hemorrhage and necrosis in the pancreas and focal hemorrhage under the epicardium were detected. Histopathologically, lymphocytic meningoencephalitis, congestion in the lung, multifocal lymphohistiocytic myocarditis, hepatitis, and pancreatitis were detected [4].

This study aimed to determine the pathogenesis of a newly emerged H5N8 AI virus (signs, lesion, histopathological changes, and duration of viral shedding from infected mule ducks) and the lethal dose 50 (LD_50_) for challenge virus in vaccine evaluation.

## Materials and Methods

### Ethical approval

All methods in the study were performed according to relevant guidelines and regulations. All experiments were carried out according to ARRIVE 2.0 guidelines and were approved by the Institutional Animal Care and Use Committee (IACUC) in the Faculty of Veterinary Medicine, Cairo University (Code: VetCU01102020217).”

### Study period and location

The study was conducted from May 2020 to December 2021 in Central Laboratory for Evaluation of Veterinary Biologics and Department of Pathology, Faculty of Veterinary Medicine, Kafr-Elsheikh University, Egypt.

### Experimental mule ducks

In this study, 55 seronegative mule ducklings (a hybrid breed originated between male Muscovy and female bikini ducks with an average size of 400–700 g) were used, which were divided into 35 ducks for LD_50_ and 20 ducks for the pathogenicity test. The ducklings were obtained from Egyptian-French Group Company by importing from France on 1-day old. Ducklings were maintained at CLEVB facilities and housed in a self-contained biosafety level three chicken isolators. Feed and water were provided *ad libitum*.

### Experimental design

In calculating the LD_50_ for local HPAI H5N8 isolates, 35 susceptible 1-week-old mule ducklings were divided into six groups (five ducks per group). Each group was inoculated with a ten-fold dilution (10^−1^–10^−6^) of local HPAI subtype H5N8. The control group was kept negative until the end of the experiment. Daily observation was performed for 10 days to report the clinical signs and mortalities, and then, LD_50_ was calculated. In addition, 20 1-week-old susceptible ducklings were inoculated with 0.1 mL of dilution containing 10LD_50_ obtained from HPAI H5N8 for the pathogenicity test. Infected ducklings were observed for 30 days, and mortalities and morbidities were recorded. Cloacal swabs were collected from infected ducks on 3^rd^, 5^th^, 7^th^, 10^th^, 14^th^, 21^st^, and 30^th^ DPI using reverse transcription polymerase chain reaction (RT-PCR) to detect viral shedding. For histopathological examination, the liver, lung, heart spleen, and brain were collected from experimental ducks on 3^rd^ and 14^th^ DPI.

### Test and procedures

#### Local HPAI subtype H5N8 virus

HPAI H5N8 virus (A/chicken/Egypt/v1526/2020) with accession no. MW600499 was obtained from the National Laboratory for Veterinary Quality Control on Poultry Production, Animal Health Research Institute, Giza.

#### Virus propagation and titration

Eighty specific pathogen-free (SPF) embryonated chicken eggs (9–11 days old) were used for virus titration and propagation [5].

### Calculation of egg infective dose/50 (EID_50_) for local HPAI subtype H5N8

The calculation was performed as described by OIE [5]. In addition, titration endpoints were calculated in accordance with a previously described method [6].

### Determination of LD_50_ [5]

Lethal dose 50 was calculated using the method of Reed and Mench [6]. Ten LD_50_ were used as the challenge virus dose for studying the pathogenesis and pathogenicity of HPAI H5N8 virus in ducklings.

### Pathogenicity test

Twenty susceptible 1-week-old ducklings were inoculated intranasally with 0.1 mL of dilution containing 10 LD_50_ (10^5^ EID_50_) from HPAI subtype H5N8. Although the previous literature recommended 100 LD_50_ (10^6^ EID_50_) titer for the pathogenicity test, we preferred 10 LD_50_ titer to keep some experimentally infected ducklings until the end of the experiment because 100 LD_50_/duck will lead to the killing of all ducklings after 5^th^ DPI based on previous data obtained after studying LD_50_. Ducklings were observed for 30 days, and all of them were diseased and died without a differential diagnosis.

### Histopathological examination

Specimens were collected from the liver, lung, heart, thymus, spleen, and brain on 3^rd^ and 14^th^ DPI, which were preserved in 10% formol saline, added with ascending grades of ethyl alcohol, and cleared in xylene. The cleared samples were embedded in paraffin and sectioned using a microtome (5-mm thickness). Then, the serial sections were stained with routine hematoxylin and eosin [7].

### Measurement of viral shedding by RT-quantitative PCR

Cloacal swabs were collected from all ducks on 3^rd^, 5^th^, 7^th^, 10^th^, 14^th^, 21^st^, and 30^th^ days post challenge and stored at −80°C until use [5].

RNA was extracted from the swabs using the QIAamp Viral RNA Mini Kit supplied from (Qiagen, Valencia, Calif., and USA) Cat. No. 52906. RNA samples were amplified using Invitrogen superscript^®^ III platinum^®^ one-step Quantitative RT-PCR Cat. No 11732-088 to investigate the presence or absence of the H5 gene of HPAI H5n8 virus following the manufacturer’s instructions using the primers and probe illustrated in Table-1. The reaction condition was conducted using the CFX 96 real-time thermal cycler from (Bio-Rad, USA).

**Table-1:** Oligonucleotide primers used in RT-qPCR: for detection of HPAI H5N8 - protein gene.

Primer	Sequence (5′ - 3′ )
Forward rh5_n8f	GGGGAATGCCCAAATATGT
Reverse rh5_n8f	TTTTGTCAATTGAGTTGACCTTATTGG
Probe	HEX-TTGGAGCTATAGCAGGTTTTATAGAGG-BHQ

RT-qPCR=Reverse transcription-quantitative polymerase chain reaction

## Results

### EID_50_ for local HPAI H5N8 virus

The local HPAI H5N8 titer was 10^9^ EID_50_/mL.

### LD_50_ for local HPAI H5N8 virus

As shown in Table-2, after inoculation with HPAI H5N8 dilution from 10^−1^ to 10^−6^ in six groups of susceptible ducklings (five ducks/groups), 10^−1^ and 10^−2^ dilutions had 100% morbidity and mortality after 4 and 5 DPI, respectively, whereas the 10^−3^ dilution leads to the killing of 80% of the infected ducklings. The 10^−4^ dilution had 20% mortality and 40% morbidity with atypical nervous signs in the form of torticollis, which continued to the end of the experiment. Moreover, the past two dilutions had low morbidity without any mortality. After recording all morbidities and mortalities during the entire observation period, the LD_50_ of the tested challenge H5N8 HPAIV was 10^4^ log_10_.

**Table-2:** LD_50_ for local HPAI H5N8 virus.

Dilution	1^st^ dpi	2^nd^ dpi	3^rd^ dpi	4^th^ dpi	5^th^ dpi	6^th^ dpi	7^th^ dpi	8^th^ dpi	9^th^ dpi	10 dpi	Mortality/total	Mortality
10^−1^												
Mortality			4	1							5/5	100%
Morbidity		4	1									
10^−2^												
Mortality				1	4						5/5	100%
Morbidity			3	4								
10^−3^												
Mortality					3	1					4/5	80%
Morbidity				4	2	1	1					
10^−4^												
Mortality						1					1/5	20%
Morbidity				1	3	2	2	2	2	2		
10^−5^												
Mortality											0/5	0%
Morbidity					1	2	2	1	1			
10^−6^												
Mortality											0/5	0%
Morbidity							1	1				

DPI=Days post-infection, LD_50_: Lethal dose 50

### Results of the inoculation of 10 LD_50_ from local HPAI H5N8 isolate in susceptible mule ducklings

**Figure-1:**
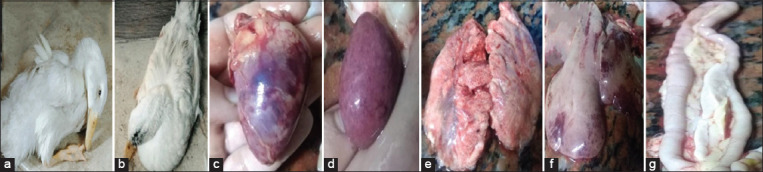
(a) Infected ducks showing depression and opisthotonus, (b) heart of infected ducks showing severe congestion and hemorrhage on coronary fat and myocardium, (c) Spleen of infected ducks showing necrotic Splenomegaly, (d) lung of infected ducks showing pneumonia with multifocal pulmonary consolidation, (e) liver of infected ducks showing necrotic patches with ecchymotic hemorrhage, and (f) pancreas of infected ducks showing necrosis and fibrosis lead to sever corrugations in the duodenum.

As shown in Table-3 and Figure-1, after the inoculation of 20 susceptible mule ducklings with 10 LD_50_ from HPAI, clinical signs began to appear on 2^nd^ DPI in the form of depression, loss of appetite, and ocular and nasal discharges. In addition, the severity of such signs increased on 3^rd^, 4^th^, and 5^th^ DPI in the form of depression, loss of appetite, grayish-white diarrhea, ocular and nasal discharge, and gasping. At 4^th^ DPI, some ducklings showed nervous signs (ataxia, head shaking, torticollis, and unusual position of the head). After 14^th^ DPI, the activity and appetite of the remaining infected ducks were improved, and the degree of severity of clinical signs decreased. At present, the percentage of morbidity decreased, reaching 10%, and whitish diarrhea was observed on 21 DPI, although all diseased ducklings were recovered on 30^th^ DPI. Mortalities were recorded on 3^rd^ DPI, and a peak was reached on 4^th^ and 5^th^ DPI. On the contrary, no mortalities were found after 6^th^ DPI. Mortalities were scored as 60% of the total number of infected ducks. Severe dehydration, cyanosed peak, reddening of the feet, and facial edema were recorded in dead ducklings. During necropsy, dead ducklings showed generalized congestion in the muscle, excess mucus in the larynx and trachea, and subepicardial hemorrhage with ecchymotic hemorrhage in coronary fat. Necrotic patches and petechial or ecchymotic hemorrhages were also observed in the liver, spleen, and pancreas with severe corrugations in the duodenum. The lung showed pneumonia with multifocal pulmonary consolidation.

**Table-3:** Morbidity, clinical signs, and mortality for infected ducklings by 10^5^ EID_50_ HPIAv H5N8.

DPI	No. of infected ducks	Morbidity Signs	Mortality	DPI	No. of infected ducks	Morbidity Signs	Mortality
1^st^ DPI	20		0	8^th^ DPI	8	5 Depression, loss of appetite, Grayish-White diarrhea	0
2^nd^ DPI	20	4 Depression, loss of appetite, ocular and nasal discharge.	0	9^th^ DPI	8	4 Depression, loss of appetite, and Grayish-white diarrhea	0
3^rd^ DPI	20	10 Depression, loss of appetite, ocular and nasal discharge. Nervous signs, Grayish-white diarrhea, and Gasping	1	10^th^ DPI	8	4 Depression, loss of appetite, and Grayish-white diarrhea	0
4^th^ DPI	19	12 Depression, loss of appetite, ocular and nasal discharge. Nervous signs, Grayish-white diarrhea, and Gasping	4	14^th^ DPI	8	1 Depressionand Grayish-white diarrhea	0
5^th^ DPI	15	8 Depression, loss of appetite, Nervous signs, Grayish-White diarrhea, ocular discharge, and Gasing.	6	21th DPI	8	1 Grayish-white diarrhea	0
6^th^ DPI	9	6 Depression, loss of appetite, Nervous signs, Grayish-white diarrhea, and ocular discharge.	1	30^th^ DPI	8	0	0
7^th^ DPI	8	5 Depression, loss of appetite, Grayish-White diarrhea, and ocular discharge.	0			Grayish-white diarrhea	

DPI=Days post-infection, EID_50_=Egg infective dose/50

Using RT-PCR, cloacal swabs were tested to detect the shedding of HPAIv H5N8 virus from infected ducklings. As shown in Table-4, on 3^rd^, 5^th^, and 7^th^ DPI, all tested cloacal swabs were positive. On 10^th^ and 14^th^ DPI, examined swabs were (3/5 and 1/5) positive, and on 21^st^ DPI, no viral shedding was detected.

**Table-4:** Results of cloacal swabs tested by RT-PCR for infected ducklings by 10^5^ EID_50_ HPIA H5N8.

DPI	No. of positive AI shedder/Total
3	5/5
5	5/5
7	5/5
10	3/5
14	1/5
21	0/5
30	0/5

DPI=Days post-infection, EID_50_=Egg infective dose/50

### Histopathological changes of the infected ducklings

Semiquantitative scoring of different lesions attributed to the challenge of influenza virus strain is illustrated in Tables-5 and 6. The early changes were represented by severe inflammatory lesions within different tissues, particularly the brain, lung, lymphoid organs, and parenchymatous organs. The pathological findings were severe in the early days post-infection (DPI) until the 14^th^ day DPI, which showed a marked decrease in lesions.

**Table-5:** Scoring of the histopathological findings of the brain, lung and liver tissues.

Days	Brain			Lung			Liver		
		
	Meningitis	Encephalitis	Malacia	Hemorrhage	Pneumonia	Necrosis	Hemorrhage	Degeneration	Necrosis
3^rd^ DPI	++++	++++	+++	++++	++++	+++	++++	+++	+++
12^th^ DPI	+	++	+	++	+	+	+	+	-

+ indicates mild lesions, ++ indicates moderate lesions, +++ indicates severe lesions and ++++ indicates severe wide-distributed lesions. DPI=Days post-infection

**Table-6:** Scoring of the histopathological findings of the kidney, spleen, and thymus.

Days	Kidney			Spleen			Thymus		
		
	Hemorrhage	Degeneration	Necrosis	Hemorrhage	Degeneration	Necrosis	Hemorrhage	Degeneration	Necrosis
3^rd^ DPI	+++	+++	++	+++	+++	++	+++	+++	++
14^th^ DPI	+	+	-	+	+	-	+	+	-

+ indicates mild lesions, ++ indicates moderate lesions, +++ indicates severe lesions and ++++ indicates severe wide-distributed lesions. DPI=Days post-infection

**Figure-2:**
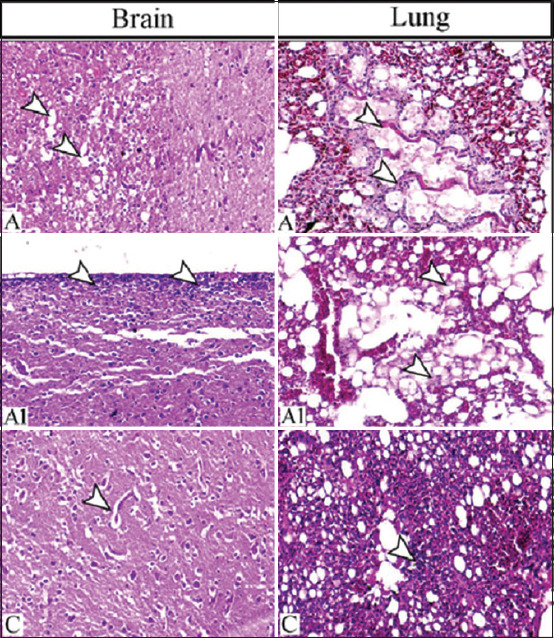
(A) Brain of challenged duck with influenza virus on the 3^rd^ DPI showing multifocal malacic foci (arrowheads) and (A1) widespread meningitis (arrowheads) and, (C) perivascular and pericellular edema (arrowhead) on the 14^th^ DPI. (A and A1) The lung showing intrabronchial hyaline membrane formation (arrowheads) on the 3^rd^ DPI and (C) marked increase of alveolar space with a focal lesion (arrowhead) on the 14^th^ DPI. H&E, 200×. A and A1 indicate the 3^rd^ DPI and (C) indicates the 14^th^ DPI. DPI=Days post-infection.

On 3^rd^ DPI, the brain primarily showed meningitis and encephalitis associated with multifocal malacic foci and gliosis either diffused or aggregated. Severe congestions of the cerebral blood vessels and widely distributed subcortical or ventricular hemorrhage were observed (Figures-2A and A1). On 14^th^ day, the brain of the challenged ducks showed decreased malacia but increased gliosis, active neuronophagia, and perivascular and pericellular edema.

In addition, the lung revealed severe pulmonary lesions in the earlier stage in the form of vascular, exudative, and necrotic changes. A marked obliteration of the parabronchial lumen with hyaline membrane and fibrinous materials, loss and break of the alveolar septa, and fibrinoid vasculitis and widespread hemorrhagic and necrotic foci were observed (Figures-2A and A1). On 14^th^ DPI, a marked increase in air capillaries and a decrease in congested blood capillaries with focal inflammatory cell aggregation were observed (Figure-2C).

**Figure-3:**
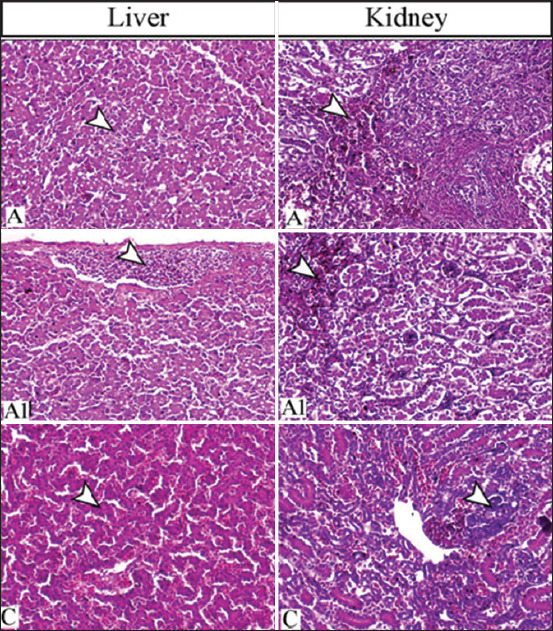
Liver of challenged duck with influenza virus on the 3^rd^ DPI showing (A) multifocal necrotic foci (arrowhead) and (A1) subcapsular hemorrhage (arrowhead) and (C) on the 14^th^ day, the liver of infected duck revealing marked restoration of normal hepatic tissues. The kidney showing (A) marked tubular degeneration mostly within the collecting tubules (arrowhead) on the 3^rd^ DPI, and (C) regenerative tubular basophilia (arrowhead) on the 14^th^ DPI. H&E, 200×. A and A1 indicate the 3^rd^ DPI and (C) indicates the 14^th^ DPI. DPI=Days post-infection.

Apart from the severe congestion of hepatic blood sinusoids and subcapsular and interstitial hemorrhages, particularly on 3^rd^ DPI, the liver of challenged ducks revealed multifocal coagulative necrosis within the hepatic tissues (Figures-3A and A1). On 14^th^ DPI, the liver tissues were within the normal limits without any viral aggregates on the nucleus or cytoplasm (Figure-3C). The kidney of a challenged bird on the 3^rd^ DPI showed severe degenerative and necrotic changes within the renal tubules, but hyaline casts were not observed in examined cases (Figure-3A1). In addition, complete recovery of the renal tubules was observed on 14 DPI (Figure-3C).

**Figure-4:**
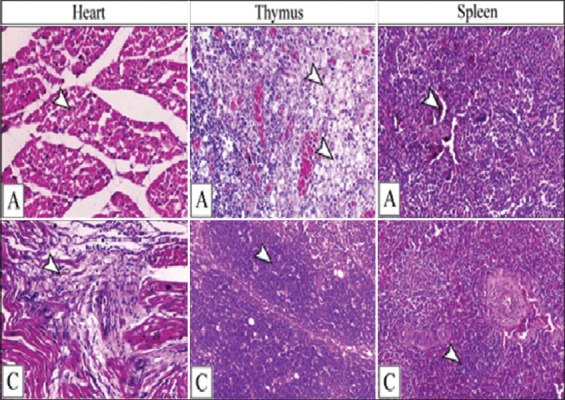
(A) The heart showing marked myolysis (arrowhead) of the cardiac muscle fibers on the 3^rd^ DPI and (C) perivascular fibrosis (arrowhead) on the 14^th^ DPI. Thymus showing (A) hemorrhage, edema and necrosis (arrowheads) and (C) restoration of the thymic compartments that are mostly filled with thymocytes (arrowhead). The spleen showing (A hemorrhage, hemosiderosis and marked depletion of the lymphoid follicle (arrowheads) on the 3^rd^ DPI and (C) restoration of the follicular lymphoid cells (arrowhead) on the 14^th^ DPI. H&E, 200×, (A) indicates the 3^rd^ DPI and (C) indicates the 14^th^ DPI. DPI=Days post-infection.

The heart of the challenged duck at 3^rd^ DPI revealed acute myocardial degeneration associated with fragmentation and myolysis of the cardiac muscle fibers, vacuolation, sub-intimal hemosiderosis, and slight interstitial mononuclear inflammatory cell infiltration (Figure-4A). Perivascular fibrosis, inflammatory cell infiltration, proliferation of the interstitial fibrous cells, and decrease in the necrobiotic changes within the muscle fibers were the prominent cardiac features on 14^th^ DPI (Figure-4C).

The lymphoid organ, including the thymus or spleen, showed severe necrotic changes associated with edema, hemorrhage, hemosiderin pigment deposition, and marked depletion of lymphoid elements, mostly on 3^rd^ DPI (Figure-4A). The features of lymphoid organ recovery were observed on the 14^th^ day, accompanied with the appearance of splenic lymphoid follicle and complete thymic compartments, which were filled with thymocytes (Figure-4C).

## Discussion

AI is considered as a great problem faced by the poultry industry in Egypt in spite of intensive vaccination. The initial outbreaks of the virus have diverged antigenically and genetically with an evident change in the pathogenicity and pathogenesis of this virus [2, 3]. Thus, this study aimed to describe the pathogenesis and pathogenicity of the newly circulating HPAI subtype H5N8 in mule ducklings in Egypt. In addition, mule ducklings were experimentally infected with HPAI H5N8 virus. The newly circulating HPAI H5N8 was propagated and titrated in ECE SPF [5], and it exhibited 10^9^ EID_50_. Furthermore, LD_50_ was calculated through the inoculation of 60 susceptible mule ducklings with 0.1 mL from 10^−1^ to 10^−6^ (10 birds/dilution) [5]. The result showed that 10^−1^ and 10^−2^ dilutions kill all infected ducklings, which was similar to a previous report [8]; that is, the HPAI A H5N8 viral infection may manifest itself as a systemic illness in commercial mule ducks with septicemic lesions, resulting in high morbidity and mortality rates of up to 100%. After recording all morbidities and mortalities for 10 days, LD_50_ was 10^4^ log_10_. The previous literature recommended 100 LD_50_ or 10^6^ EID_50_ to investigate the pathogenesis and pathogenicity of tested AI virus [9–11]. However, in this study, 10 LD_50_ (10^5^ EID_50_) was selected to keep some living ducklings until the end of the experiment. Twenty susceptible ducklings were inoculated intranasally with 0.1 mL dilution containing 10 LD_50_ of the newly emerged HPAI H5N8, and morbidities with signs and mortalities were recorded until 30 DPI. The obtained results revealed that the clinical signs began on the 2^nd^ 2 DPI with a morbidity rate reaching 20%, which was similar to that obtained by a previous study [12]. Lethargy, loss of appetite, ocular and nasal discharge, and grayish-white diarrhea accompanied by nervous manifestations in the form of head shaking, torticollis, and abnormal position of the head with an average of 30% were the primary clinical signs, which were observed after 3^rd^ and 4^th^ DPI. These results were consistent with other experimental infections of ducks with HPAI clade 2.3.4 that showed severe neurologic signs [9–14]. Mortalities reached the peak after 4^th^ and 5^th^ DPI with average 50%, which is similar to previously reported data [11]; that is, ducks challenged by HPAI H5N8 died between 4^th^ and 6^th^ DPI with an average 60%. In addition, no mortalities were found after 6^th^ DPI, which is consistent with a previous study [15]. On the contrary, Haider *et al*. [16] reported 47% and 61% mortality rates in ducks from Bangladesh and India, respectively. Dead ducklings showed severe dehydration, cyanosed peak, reddening of the feet, and facial edema. These results were consistent with those obtained by Lean *et al*. [17]. Gross lesions were summarized in the form of necrosis, and petechiation or ecchymotic hemorrhage was also observed in the liver, spleen, and pancreas with severe corrugations in the duodenum. The lung showed pneumonia with multifocal pulmonary consolidation. The infected heart showed subepicardial hemorrhage with ecchymotic hemorrhage on coronary fats. These results are consistent with that found by Banyai *et al*. [4] and Jackwood *et al*. [12]. In detecting viral shedding from infected ducks, cloacal swabs were collected on the 3^rd^, 5^th^, 7^th^, 10^th^, 14^th^, 21^st^, and 30^th^ DPI, and the virus was detected using RT-PCR. As shown in Table-4, all the infected ducklings shed the virus from its gut on 3^rd^, 5^th^, and 7^th^ DPI, which is consistent with a previous study [11]; that is, infected ducks can release the virus through cloaca after the 2^nd^ DPI, and high levels of viral shedding were observed for Pekin ducks, which peaked at around 5 DPI. At present, the number of ducklings with viral shedding gradually decreased (3/5 and 1/5 on 10^th^ and 14^th^ DPI, respectively). These results are consistent with that found by Jackwood *et al*. [12], and Spackman *et al*. [15], who proved that no viral shedding occurred after 14^th^ DPI. The histopathological examination of the liver, lung, heart, thymus, spleen, and brain from experimental ducks on 3^rd^ and 14^th^ DPI revealed that the brain of challenged ducks showed multifocal malacic foci and widespread meningitis on 3^rd^ DPI, which was consistent with that obtained by Dinev *et al*. [8] and perivascular pericellular edema on 14^th^ DPI. The lung showed intrabronchial hyaline membrane formation, consolidation of the lung, and intra-alveolar fibrinous exudate on the 3^rd^ day and marked increase of alveolar space with a focal lesion on the 14^th^ DPI, which was consistent with that of Kim *et al*. [18]. In addition, the liver of challenged ducks with H5N8 HPAIV on 3^rd^ DPI showed multifocal necrotic foci, vacuolar degeneration, and subcapsular hemorrhage, which was consistent with that found by Nooruzzaman *et al*. [19]. On the one hand, on 14^th^ DPI, the liver of infected ducks revealed marked restoration of normal hepatic tissues. The kidney showed marked tubular necrosis mostly within the collecting tubules on the 3^rd^ DPI, which was consistent with that reported by Nooruzzaman *et al*. [19]. On the other hand, on 14^th^ DPI, the kidney showed less tubular degeneration and regenerative tubular basophilia. The heart showed severe intermyofibrillar edema, moderate-to-massive hemorrhages, and marked myolysis of the cardiac muscle fibers on 3^rd^ DPI, which was consistent with that of Dinev *et al*. [8], whereas perivascular inflammation was associated with fibroblastic cell proliferation on 14^th^ DPI. Moreover, the thymus showed hemorrhage edema and necrosis on 3^rd^ DPI and restoration of the thymic compartments that were mostly filled with thymocytes on 14^th^ DPI. The spleen showed necrosis, hemosiderosis, and marked depletion of the lymphoid follicle on 3^rd^ DPI, which was consistent with that reported by Jackwood *et al*. [12] and Caliendo *et al*. [20], and restoration of the follicular lymphoid cells on 14^th^ DPI.

## Conclusion

In this study, the newly circulating HPAI subtype H5N8 became more virulent when compared with isolated strains based on the previous studies, particularly for imported mule ducks that originated from non-vaccinated breeder ducks. Subsequently, the vaccination of ducks on 1-day old and quarantine measures are necessary to prevent infection by this virus. Thus, continuous and rapid evolution of those AI viruses is recommended, which necessitates reviewing and monitoring the pathogenesis for updating circulating AI strains.

## Authors’ Contributions

MMA, WS, and SAN: Conducted the experiment and drafted the manuscript. MMA, AM, EF, WS, and SAN: Designed the study and followed up the experiment and critically reviewed the manuscript. WS: Performed histopathological examinations. MMA, AM, EF, and SAN: Participated in study design and followed up on the practical work. All authors have read and approved the final manuscript.
